# Effect of Donor-Acceptor Vertical Composition Profile on Performance of Organic Bulk Heterojunction Solar Cells

**DOI:** 10.1038/s41598-018-27868-2

**Published:** 2018-06-22

**Authors:** Sheng Bi, Zhongliang Ouyang, Shoieb Shaik, Dawen Li

**Affiliations:** 1 Key Laboratory for Precision and Non-traditional Machining Technology of the Ministry of Education, Dalian University of Technology, No. 2 Linggong Rd, Dalian, 116024 P.R. China; 2 Department of Electrical and Computer Engineering, Center for Materials for Information Technology, The University of Alabama, Tuscaloosa, AL 35487 USA

## Abstract

In organic bulk heterojunction solar cells (OSCs) donor-acceptor vertical composition profile is one of the crucial factors that affect power-conversion efficiency (PCE). In this simulation study, five different kinds of donor-acceptor vertical configurations, including sandwich type I and type II, charge transport favorable, charge transport unfavorable, and uniform vertical distribution, have been investigated for both regular and inverted OSC structures. OSCs with uniform and charge transport favorable vertical composition profiles demonstrate the highest efficiencies. High PCE from charge transport favorable configuration can be attributed to low recombination because of facilitated charge transport in active layer and collection at electrodes, while high PCE from uniform structure is due to sufficient interfaces for efficient exciton dissociation. OSCs with sandwich and charge transport unfavorable structures show much lower efficiencies. The physical mechanisms behind simulation results are explained based on energy band diagrams, dark current-voltage characteristics, and comparison of external quantum efficiency. In conclusion, experimental optimization of vertical composition profile should be directed to either uniform or charge transport favorable vertical configurations in order to achieve high-performance OSCs.

## Introduction

Polymer-fullerene bulk heterojunction organic solar cells (OSCs) have become one of the research focuses worldwide by showing great promise as a green, flexible and low-cost renewable energy source^1–11^. In recent years, the record efficiency has been frequently broken through^12–14^. However, regardless of the progress made on energy-conversion efficiency, the research on the device optimization and fundamental understanding of the physical mechanisms is still on the way.

Morphology control of bulk heterojunction active layer is one of key factors for improving performance of OSCs. It is well known that nanoscale interpenetrating donor-acceptor network increases interfacial areas, thereby enhancing exciton dissociation at the polymer-fullerene interface, while the vertical distribution of each phase in the blend film influences charge transport and collection to the electrodes. The blend morphology of electron-donating polymer and fullerene acceptor is sensitive to processing conditions. There have been substantial efforts in process engineering to attain desired morphology in polymer-fullerene bulk heterojunction, particularly P3HT-PCBM system^15–19^. With optimization of solution processing conditions, uniform morphology in the lateral direction could typically be obtained. However, due to the sensitivity on surface energy and processing conditions, various P3HT-PCBM vertical composition profiles have been observed. For example, some research groups demonstrated that the difference of surface energy and interactions between fullerene and substrate lead to a PCBM aggregation near the ITO anode while P3HT stays close to the cathode in the regular OSC structure^15,20^. Such vertical phase separation has been taken as an advantage for OSCs with inverted structure^21^. On the other hand, van Bavel *et al*. discovered an opposite vertical structure of P3HT tending to accumulate next to the ITO anode and PCBM to the cathode^22^. Jay Guo group utilized a gas-permeable cover layer to manipulate surface energy and attain an almost uniform vertical distribution of P3HT/PCBM components inside blend^23^. Interestingly, another vertical configuration, i.e., sandwich structure, was reported by Z. Sun and his colleagues. In such sandwich vertical composition profile, P3HT-rich layer were found in the middle of the active layer and sandwiched by PCBM-rich layers near both anode and cathode contacts^24^. Various schemes for morphology control and characterization in the vertical direction have also been reported^20,25–31^. In addition to the experiments, different simulation methods are utilized to study the effect of device morphology on the device performance. Watkins *et al*. applied dynamical Monte Carlo (DMC) Modelling to the PFB/F8BT system, finding that the internal quantum efficiency is strongly sensitive to the morphological phase separation^32^. Huang adopted molecular dynamics (MD) simulations to investigate the P3HT/PCBM system, concluding that the microstructure of polymer/fullerene blends has a key impact on the device performance^33^. Ji *et al*. reviewed various methods that characterize the morphology-efficiency relationship of polymer solar cells, including the DMC method, MD method and dissipative particle dynamics (DPD) method^34^. In a previous work, the P3HT/PCBM system have been researched through both experiments and simulation, analyzing the vertical configurations through comparison of absorption, quantum efficiency, recombination, resistance and current leakage^35^. The diverse distribution of P3HT and PCBM in the vertical direction results in different capability of charge carrier transport in the active layer and collection at the electrodes, thereby affecting device performance.

In this work, commercial simulator Semiconducting Thin Film Optics Simulation software (SETFOS)^36–38^ from Fluxim AG was used to perform all the modelling of P3HT/PCBM solar cells. SETFOS offers an easy-to-use graphical user interface, which allows building the vertical structures of the solar cells layer by layer, specifying all the optical/electrical parameters, performing the actual simulation and collecting/plotting the results. The physical principles behind SETFOS are the coupling of the photonic absorption and the drift-diffusion models in organic semiconducting devices. In the simulation of optical model, transfer matrix approach^39^ is utilized to calculate optical properties. Specifically, the overall absorption profiles of thin multilayer structures are derived by considering the complex refractive index $\bar{n}=n+i\kappa$ of each layer^35^. In cases that one layer consists of two materials, the weighted average of their complex refractive indices has been input as parameter. In addition to the absorption, the light scattering at interfaces is also taken into account, adopting the scalar scattering model developed by Santbergen^40^ and Lanz^41^. The AM 1.5 sun spectrum is input as the light source for the calculation of the absorption profile, which will be transferred to the built-in electrical model for evaluation of the charge transport and electronic outputs. In the electrical model, the production and loss of the electrons and holes are described by the space-charge continuity equations (1) and (2), where *n*, *p*, *J*_*n*_, *J*_*p*_, *R*_*L*_ and *G* are the electron concentration, hole concentration, electron current density, hole current density, recombination rate and generation rate, respectively. The current densities are given by equations (3) and (4), with *μ*_*n*_, *μ*_*p*_, *E*, *k*_*B*_ and *T* being the electron mobility, hole mobility, electrical field, Boltzmann constant and absolute temperature. The recombination is believed to be a bimolecular process in organic semiconductors. Therefore, the recombination assumes the Langevin type in equation (5), where *η*_*L*_, *ε*_0_ and *ε*_*r*_ represent the Langevin recombination efficiency, vacuum and relative dielectric constants, respectively. The generation rate is expressed in equation (6) as the product of the exciton concentration *N*_*ex*_ and the optical charge generation efficiency *g*_*opt*_. The exciton concentration *N*_*ex*_ is determined by the optical absorption profile *Q*, which comes from the optical model mentioned above, and the exciton energy *hc*/*λ*. Equation (7) is the Poisson’s equation relating the electric field *E*, electrical potential *φ* and space charge, which includes free charge carrier densities and localized ionized dopant densities *N*_*D*_ and *N*_*A*_. This study does not consider doping of any kinds so no such parameter is given. In the simulation, energy levels have been assumed to be sharp, characterized by unique values of the HOMO and LUMO levels for active materials. At the electrodes, ohmic boundary condition has been adopted and workfunctions of the electrodes need to be specified. As a result, the charge carrier densities at electrodes can be computed through equations (8) and (9), where *ϕ* is the workfunction and *N*_0_ is the density of states (1.0 × 10^27^ *m*^−3^, default value in the software). These coupled equations are solved by the SETFOS simulator to give the *J-V* curves, fill factors, *Voc*s, *Jsc*s and power conversion efficiencies of the studied P3HT/PCBM systems.

1 $$\frac{\partial n}{\partial t}=\frac{\mathop{\nabla }\limits^{\rightharpoonup }\mathop{{J}_{n}}\limits^{\rightharpoonup }}{e}-{R}_{L}+G$$

2 $$\frac{\partial p}{\partial t}=-\frac{\mathop{\nabla }\limits^{\rightharpoonup }\mathop{{J}_{p}}\limits^{\rightharpoonup }}{e}-{R}_{L}+G$$

3 $${J}_{n}=en{\mu }_{n}E+{\mu }_{n}{k}_{B}T\frac{\partial n}{\partial x}$$

4 $${J}_{p}=ep{\mu }_{p}E+{\mu }_{p}{k}_{B}T\frac{\partial p}{\partial x}$$

5 $${R}_{L}={\eta }_{L}({\mu }_{n}+{\mu }_{p})np\frac{e}{{\varepsilon }_{0}{\varepsilon }_{r}}$$

6 $$G={g}_{opt}{N}_{ex}={g}_{opt}\frac{Q}{hc/\lambda }$$

7 $${\varepsilon }_{0}{\varepsilon }_{r}\mathop{\nabla }\limits^{\rightharpoonup }\mathop{E}\limits^{\rightharpoonup }=-\,{\varepsilon }_{0}{\varepsilon }_{r}{\nabla }^{2}\phi =e(p-n+{N}_{D}-{N}_{A})$$

8 $${p}_{electrode}={N}_{0}\cdot \exp (\frac{\varphi -HOMO}{{k}_{B}T})$$

9 $${n}_{electrode}={N}_{0}\cdot \exp (\frac{LUMO-\varphi }{{k}_{B}T})$$

In this study, various P3HT-PCBM vertical configurations in active layer were modelled and systematically investigated for both regular and inverted OSC structures. P3HT-PCBM blending system is adopted as a benchmark here because this donor-acceptor blend has been extensively studied and reported, which provides massive literature database for comparison. After comparing the performance of OSCs with different vertical configurations, we further investigated the physical mechanisms behind simulation results using energy band diagrams, dark current-voltage characteristics, and comparison of external quantum efficiencies.

**Figure 1:**
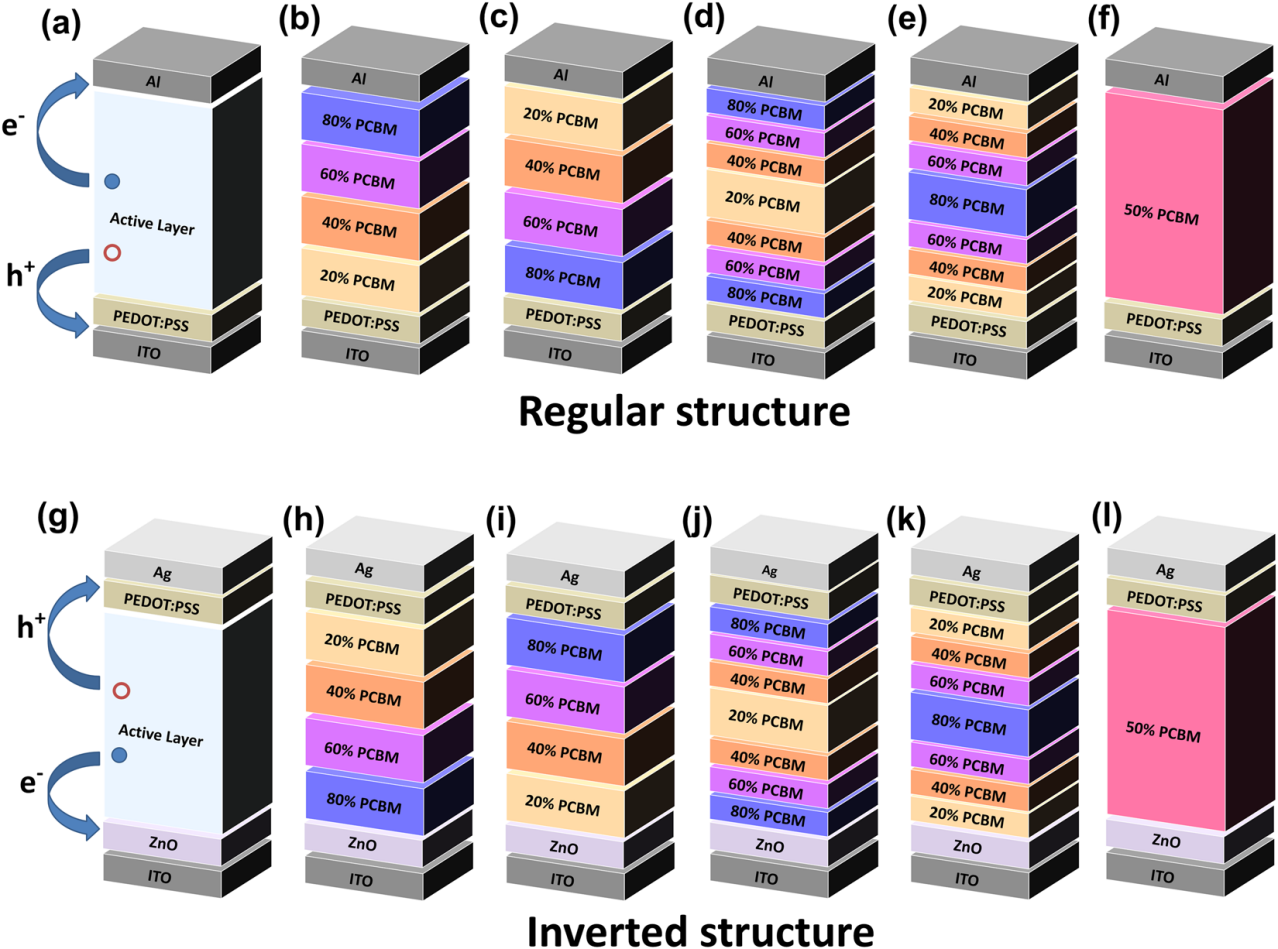
Schematic of (**a**) regular OSC architecture with indication of electrons and holes transport directions, and various vertical composition profiles as (**b**) charge transport favorable, (**c**) unfavorable, (**d**) sandwich I, (**e**) sandwich II, (**f**) uniform configuration. And schematic of (**g**) the inverted OSC structure, and different vertical configurations as (**h**) charge transport favorable, (**i**) unfavorable, (**j**) sandwich I, (**k**) sandwich II, and (**l**) uniform active layer.

**Figure 2:**
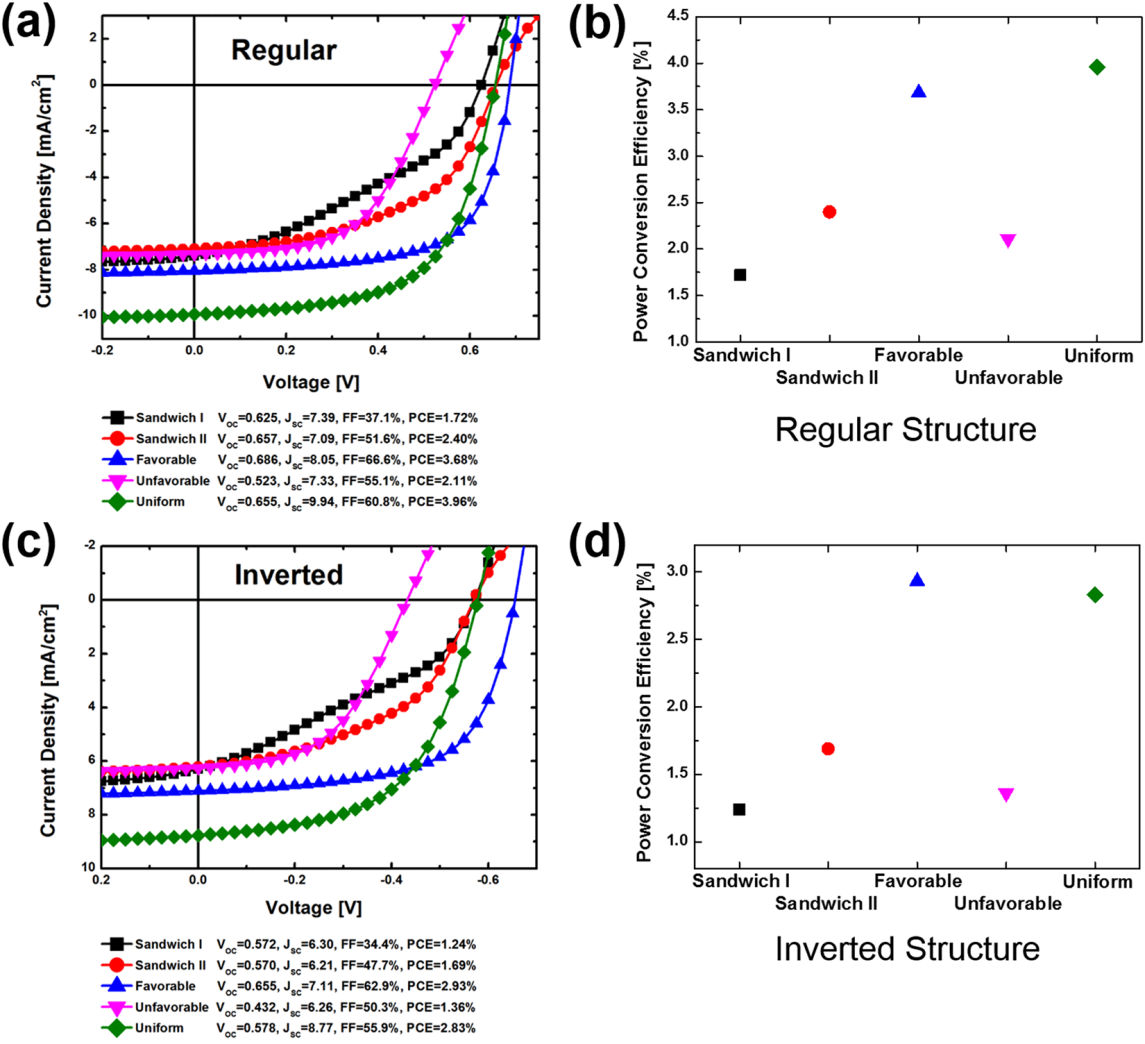
Simulated J-V characteristics of (**a**) regular structure and (**b**) resulting PCEs, and (**c**) inverted solar cells and (**d**) extracted PCEs, with sandwich I, sandwich II, charge transport favorable, unfavorable and uniform vertical configurations. The values of important parameters such as *Voc*, *Jsc*, *FF* and PCE are also listed.

Figure 1(a) illustrates regular OSC structure with electrons moving toward Al cathode and holes transporting to ITO anode through PEDOT:PSS hole transport layer. Figure 1(b–f) show schematics of five different vertical configurations, including charge transport favorable, charge transport unfavorable, sandwich I, sandwich II, and P3HT/PCBM equally distributed uniform structures. Since electrons transport in PCBM and are collected at Al cathode and holes transport in P3HT and are collected at ITO anode in regular OSC architecture, PCBM-rich layer close to the Al cathode facilitates electron transport and collection, while P3HT-rich layer near the ITO anode benefits hole transportation and collection. In charge transport favorable structure (Fig. 1(b)) the weight percentage of PCBM increases monotonically from ITO anode side to Al cathode end as 20%, 40%, 60% and 80%, in which P3HT weight percentage decreases accordingly. The charge transport unfavorable structure possesses a completely reversed PCBM/P3HT distribution as compared to the favorable one. Through the charge transport unfavorable configuration, the weight ratio of PCBM decreases continuously from 80% to 20% in the active layer, while concentration of P3HT increases from 20% to 80% accordingly from ITO anode side to the Al cathode end. Each constructing layer of various PCBM percentages has thickness of 20 nm with total active layer thickness of 80 nm. There also exist two kinds of sandwich structures. In sandwich I configuration (Fig. 1(d)), a layer of 20% PCBM is introduced in the middle of active layer to represent the P3HT-rich layer, and the concentration of PCBM gradually increases toward both anode and cathode ends, i.e. 40% and 60% PCBM layers are placed outwards subsequently with 80% PCBM aggregation near the electrodes. The sandwich II configuration follows an opposite distribution, in which 80% PCBM-rich layer is sandwiched by layers with gradually reduced PCBM percentage, that is, 60% PCBM layer followed by 40% PCBM layer in series till intense P3HT aggregation layers are placed at both ends of the active layer. For sandwich structure, the centered layer has thickness of 20 nm, while each outer layers serving as the concentration gradient of PCBM have thickness of 10 nm. As for the uniform structure, 50% PCBM and 50% P3HT are evenly mixed and distributed across the entire active layer in both lateral and vertical directions. All the structures described above have the same total active layer thickness of 80 nm. Based on typical literature reports, the thicknesses of Al, PEDOT:PSS and ITO are taken to be 100 nm, 40 nm, 120 nm, respectively. In addition to the investigation on regular organic polymeric solar cells, we also study the effect of vertical composition profile on performance of OSCs with inverted structures. As illustrated in Fig. 1(g), in inverted organic bulk heterojunction solar cells, holes move upward through PEDOT:PSS hole transport layer and are collected at Ag anode, while electrons move downward through ZnO electron transport layer and are collected at ITO cathode. The difference between regular and inverted organic bulk heterojunction solar cells mainly lies in the opposite charge transport directions resulted from different transport layers and charge collecting electrodes. Figure 2(h–l) shows the different vertical composition profiles in inverted OSC structure. The charge transport favorable and unfavorable configurations are opposite to those in regular solar cell structure because of the opposite charge transport directions.

In the device modeling, the work functions of ITO, PEDOT:PSS, and Al are 4.5 eV, 5.0 eV and 4.3 eV, respectively, which are used in most literatures as standard values. The lowest unoccupied molecular orbital (LUMO) and the highest occupied molecular orbital (HOMO) energy levels of P3HT are 3.3 eV and 5.3 eV, respectively, and PCBM has LUMO energy level of 4.0 eV and HOMO energy level of 6.1 eV. The dielectric constants of P3HT and PCBM are 3.0 and 3.9, respectively^42–45^. For each specific layer, both the dielectric constant and the refractive index are linearly weighted based on the ratio of each component^46,47^. Other physical parameters used in SETFOS modeling, including electron and hole mobility, free charge carrier generation efficiency, and Langevin recombination efficiency, are summarized in Table 1^48–53^. As input parameters, the values of all these electronic properties are taken from literature reports and variations in trend as a function of PCBM percentage are justified based on material and device physics underneath.

**Table 1:** Electronic parameters of P3HT/PCBM blend at different PCBM percentages.

Parameter	20% PCBM	40% PCBM	50% PCBM	60% PCBM	80% PCBM
Dielectric Constant	3.18	3.36	3.45	3.54	3.72
Electron mobility (cm^2^V^−1^s^−1^)	7 × 10^−5^	7 × 10^−4^	1 × 10^−3^	4 × 10^−3^	3 × 10^−2^
Hole mobility (cm^2^V^−1^s^−1^)	4 × 10^−3^	1 × 10^−3^	7 × 10^−4^	4 × 10^−4^	9 × 10^−5^
Optical charge generation efficiency	0.4	0.8	0.8	0.8	0.25
Langevin recombination efficiency	0.1	0.2	0.2	0.2	0.1

It is anticipated that electron mobility increases as PCBM percentage increases, while hole mobility increases as PCBM loading ratio decreases, which is equivalent to increase of P3HT concentrations. Since PCBM acceptor carries electrons, the increase of PCBM leads to an enhanced electron transport. Similarly, the increase of P3HT donor results in enhancement of hole mobility. The mobility values adopted in Table 1 match well with experimental results reported in literature^52,53^. In terms of charge generation efficiency, typically 1:1 weight ratio of P3HT and PCBM is used to obtain nanoscale interpenetrating film morphology for efficient exciton dissociation and leads to a high optical charge generation efficiency^48–51^. With this consideration, the charge generation efficiencies of blends with 40–60% PCBM are reasonably set to be higher than those in 20% and 80% PCBM active layers. Also the charge generation efficiency in 20% PCBM (80% P3HT) is set to be slightly higher than that in 80% PCBM active layer because P3HT is the main material that absorbs sunlight and produces electron-hole pairs. It is expected that Langevin recombination efficiency has similar dependence on PCBM percentage as charge generation efficiency. According to the equation (5), the Langevin recombination efficiency depends on charge carrier mobility and concentrations^54^.

Since there is no big difference in sum of electron and hole mobility (*μ*_*n*_ + *μ*_*p*_) as PCBM percentage varies, the Langevin recombination efficiency mainly depends on the generated charge carrier concentrations *np*. Because more electrons and holes are generated in 40% PCBM, 50% PCBM and 60% PCBM active layers, a higher $np$ value results in the higher Langevin recombination efficiency. Therefore, the Langevin recombination efficiencies at 40–60% PCBM levels are reasonably set to be greater than those in 20% PCBM and 80% PCBM layers. With the listed parameter values in Table 1 as input, the simulated OSC efficiencies are comparable with reported values from experiments^55,56^. Once these electronic parameters at different PCBM percentages are validated, organic bulk heterojunction solar cells with different vertical composition profiles are investigated.

Figure 2(a) shows the simulated current density versus voltage (J-V) characteristics of regular OSC structure with different vertical configurations. The power-conversion efficiencies (PCEs) extracted from these J-V curves are summarized in Fig. 2(b). Similarly, Fig. 2(c,d) show J-V characteristics and extracted PCE of inverted solar cells, respectively. Solar cells with uniform and charge transport favorable composition profiles for both regular and inverted structures demonstrate much higher efficiencies than those with charge transport unfavorable and sandwich configurations. J-V curves from the uniform and charge transport favorable vertical configurations show square-like shape, while the poorest device performance from sandwich I configuration are resulted from severe S-shaped J-V curves.

In order to gain the insight of the effect of vertical configurations on the efficiency, important output characteristics, including open-circuit voltage (*V*_*OC*_), short-circuit current density (*J*_*SC*_), and fill factor (*FF*), are also examined. The values of these extracted parameters are provided in Fig. 2(a,c) for regular and inverted solar cells, respectively. Of all different vertical configurations, the highest *Voc* occurs in OSCs with charge transport favorable vertical configuration while the lowest one appears in those with the charge transport unfavorable composition profile. Since *Voc* is determined by the recombination of charge carriers, the smooth charge transport from favorable structure results in less recombination, leading to larger open-circuit voltage. While the charge transport unfavorable structure is anticipated to has the largest charge carrier recombination, consequently resulting in the lowest *Voc*. In terms of the short-current density, the uniform configuration has the highest value followed by the charge transport favorable structure. The equal amount of mixture of P3HT donors and PCBM acceptors results in the largest interface area for efficient exciton dissociation. The charge transport favorable configuration facilities both electron and hole transport in the active layer and charge carrier collection at the electrodes, consequently leading to less recombination and greater output carriers, thus larger *Jsc*. As for the fill factor, OSCs with charge transport favorable configuration shows the highest fill factor followed by the uniform configuration. Sandwich and charge transport unfavorable structures have relative low fill factor, particularly, the sandwich I structure has the lowest fill factor. It is widely known that the fill factor mainly depends on charge carrier recombination, series resistance and shunt resistance. Series resistance usually comes from contact and material resistance, while shunt resistance results from current leakage, i.e. the charge carriers move to the opposite direction and are collected by the wrong electrode. Low recombination, small series resistance and large shunt resistance lead to a high fill factor.

**Figure 3:**
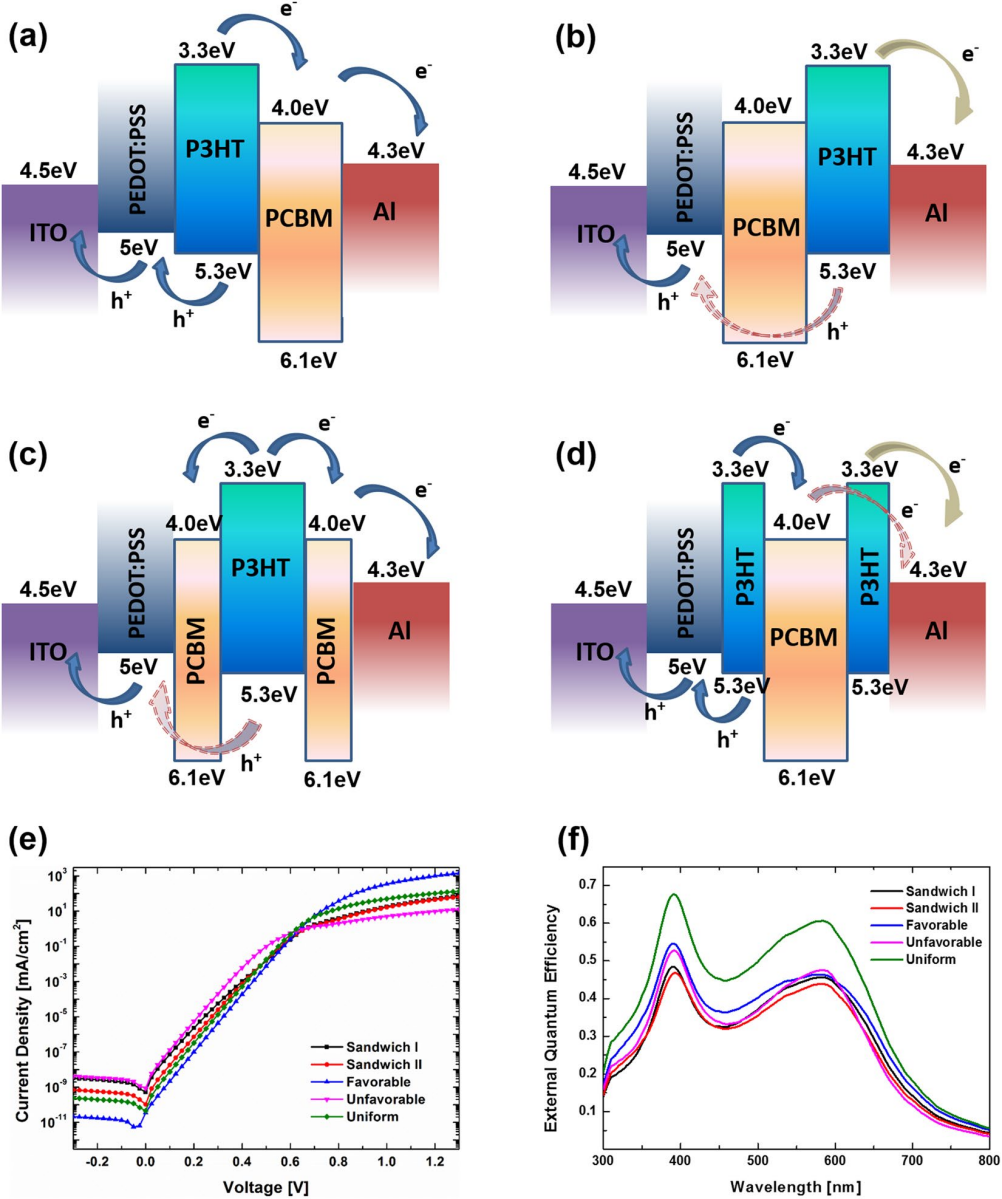
Energy band diagram of (**a**) charge transport favorable/uniform, (**b**) charge transport unfavorable, (**c**) sandwich I, (**d**) sandwich II configurations for OSCs with regular structure, (**e**) simulated dark J-V curves and (**f**) external quantum efficiencies.

The charge transport, recombination and current leakage could be explained using energy band diagrams as shown in Fig. 3. The charge transport favorable and uniform vertical configurations facilitate electrons movement at LUMO energy levels toward Al cathode and holes movement at HOMO levels to ITO anode (Fig. 3(a)), resulting in low charge carrier recombination and no leakage current, thereby high open-circuit voltage and fill factor. As shown in Fig. 3(b–d), charge transport unfavorable and sandwich structures always have energy barriers for electrons transport and collection at cathode and holes at anode, consequently leading to high recombination and low *Voc* and fill factor. Particularly, electrons at the LUMO energy level in sandwich I structure could freely move toward both Al cathode and ITO anode. Those electrons collected at ITO anode cause leakage current, severely reducing the shunt resistance thereby yielding the lowest fill factor. The leakage currents can be further verified by the simulated dark J-V characteristics as shown in Fig. 3(e), in which charge transport favorable configuration demonstrates the lowest leakage current while charge transport unfavorable and sandwich I structures show the largest leakage currents^57–59^. In addition, the variations of short-current density for different vertical composition profiles matches well with external quantum efficiency (EQE). As shown in in Fig. 3(f), the highest EQE attributing to the largest current density is from the uniform configuration followed by charge transport favorable structure. Based on the equation of power-conversion efficiency $\,{\rm{PCE}}={V}_{OC}{J}_{SC}\,FF/{P}_{in}$, where *P*_*in*_ is the input power, PCE is determined by the product of *Voc*, *Jsc*, and *FF*. The increase of any of these parameters will improve the PCE. OSCs with uniform and charge transport favorable vertical composition profiles demonstrate large values in all these three categories, leading to high PCEs. In contrast, all these three electronic properties of OSCs with charge transport unfavorable and sandwich structures fall in low values, particularly sandwich I has the smallest fill factor, resulting in the lowest PCE.

**Figure 4:**
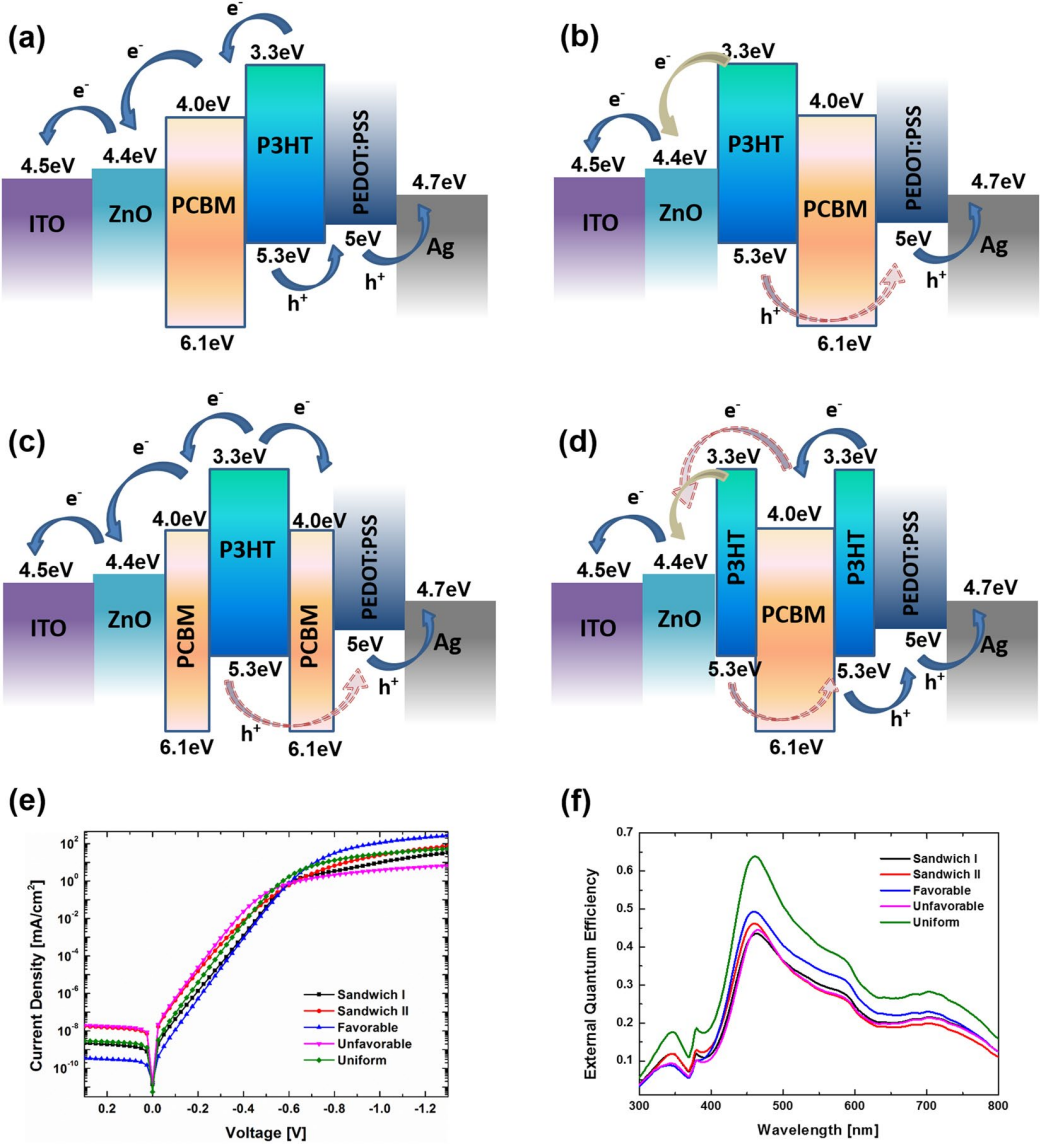
Energy band diagram of (**a**) charge transport favorable/uniform, (**b**) charge transport unfavorable, (**c**) sandwich I, (**d**) sandwich II configurations for OSCs with inverted structure, (**e**) simulated dark J-V curves and (**f**) external quantum efficiencies.

Similar analysis can be applied to the inverted solar cells. The charge transport, recombination and current leakage could also be explained using energy band diagrams and verified by simulated dark J-V characteristics and external quantum efficiency curves, as shown in Fig. 4. It is safe to conclude that uniform and charge transport favorable vertical composition profiles are desirable to achieve high power-conversion efficiency in organic polymeric bulk heterojunction solar cells.

In conclusion, the vertical configuration of organic bulk heterojunction solar cells plays a critical role in improving power-conversion efficiency. The quality of vertical pathway directly determines the performance of solar cells. The vertical distribution of P3HT donors and PCBM acceptors not only has an impact on charge carrier transportation, but also influence exciton dissociation. A uniform distribution of 50% donors and 50% acceptors results in the most efficient exciton separation due to the largest interfacial areas. On the other hand, smooth charge carrier transportation from charge transport favorable vertical configuration causes less recombination before charge carriers reach electrodes. In this study, different vertical composition profiles have been modeled for both regular and inverted OSC architectures. The results show that charge transport favorable and uniform configurations always give the highest efficiencies, while sandwich and charge transport unfavorable structures tend to have poor performance. In addition, the analysis of charge carrier transport, recombination, and leakage current from energy band diagrams, dark J-V characteristics and comparison of external quantum efficiencies provides an in-depth understanding of physical mechanisms behind the effect of different vertical composition profiles of active layer on performance of organic bulk heterojunction solar cells.
